# Ambulatory Arterial Stiffness Index Is Higher in Hypertensive Patients with Chronic Kidney Disease

**DOI:** 10.1155/2012/178078

**Published:** 2012-05-21

**Authors:** Ronaldo Altenburg Gismondi, Mario Fritsch Neves, Wille Oigman, Rachel Bregman

**Affiliations:** ^1^Department of Clinical Medicine, Rio de Janeiro State University, Rio de Janeiro, RJ, Brazil; ^2^Departamento de Clínica Médica, Hospital Universitario Pedro Ernesto, Avenue 28 de Setembro 77, Sala 329, Vila Isabel, 20551-030 Rio de Janeiro, RJ, Brazil; ^3^Nephrology Division, Rio de Janeiro State University, Rio de Janeiro, RJ, Brazil

## Abstract

Ambulatory arterial stiffness index (AASI) is a parameter obtained from ambulatory blood pressure monitoring (ABPM) that correlates with clinical endpoints. The aim of this study was to compare AASI in nondiabetic hypertensive patients with and without chronic kidney disease (CKD). Subjects with systemic arterial hypertension (SAH, *n* = 30) with normal renal function, aged 40 to 75 years, were compared to hypertensive patients with CKD (*n* = 30) presenting estimated glomerular filtration rate (eGFR) <60 mL/min by MDRD formula. ABPM was carried out in all patients. In CKD group, eGFR was 35.3 ± 2.8 ml/min. The mean 24-hour systolic and diastolic blood pressure (BP) was similar in both groups. AASI was significantly higher in CKD group (0.45 ± 0.03 versus 0.37 ± 0.02, *P* < 0.05), positively correlated to age (*r* = 0.38, *P* < 0.01) and pulse pressure (*r* = 0.43, *P* < 0.01) and negatively correlated to nocturnal BP fall (*r* = -0.28, *P* = 0.03). These findings indicate the presence of stiffer vessels in CKD hypertensive patients.

## 1. Introduction

Epidemiological and observational studies indicate hypertension as a major cause of chronic kidney disease (CKD) [1]. In fact, hypertension and CKD are strongly connected because hypertension is both a cause and a consequence of CKD [2]. Hypertensive patients with CKD present higher morbidity and mortality rates when compared with those with normal renal function [3, 4]. Additionally, high blood pressure is a predictor of decline of glomerular filtration rate (GFR), and conversely, adequate blood pressure control contributes to preserve renal function [5–7].

Vascular changes are commonly observed in CKD patients, including reduced arterial elasticity observed in patients with end-stage renal disease [8, 9]. Fibroelastic intimal thickening, increased extracellular matrix, enhanced collagen density, and vascular calcification seem to contribute to stiffer arteries in CKD patients [10, 11]. It has been demonstrated that vascular stiffness may predict adverse cardiovascular outcomes [12, 13]. Current gold standard for vascular stiffness evaluation is the pulse wave velocity (PWV) which requires complex equipment and therefore is not commonly used in clinical practice [14]. Recently, a new parameter named ambulatory arterial stiffness index (AASI) was proposed for this evaluation [15, 16]. This index is derived from the regression slope of the diastolic on systolic blood pressure, using all of the readings during ambulatory blood pressure monitoring (ABPM).

It has already been reported that AASI presents good correlation with target organ damage and glomerular filtration rate (GFR) in essential hypertension [17–20]. Moreover, AASI may also correlate with cardiovascular events and mortality [21, 22]. On the other hand, there have been few studies investigating this index in hypertensive patients with different stages of CKD. The aim of our study was to evaluate the AASI in nondiabetic hypertensive patients with CKD and in those with normal renal function.

## 2. Methods

A case-control study involving 60 consecutive patients with primary hypertension was carried out in our institution. Thirty patients were recruited from the CKD outpatient clinic. Hypertensive patients with CKD, aged 40 to 75 years and estimated glomerular filtration rate (eGFR) <60 mL/min by the Modification of Diet in Renal Disease (MDRD) equation [23], were included (CKD group). Other 30 patients matched by age (±2 years) and gender with the CKD patients were selected from the hypertension outpatient clinic at the same institution. These patients presented systemic arterial hypertension (SAH group) and serum creatinine less than 1 mg/dL. Exclusion criteria were diabetes mellitus, hypertriglyceridemia (>400 mg/dL), urinary albumin-to-creatinine ratio (UACR) >1000 mg/g, acute renal failure, renal replacement therapy, regular use of anti-inflammatory drugs, and history of myocardial infarction or cerebrovascular disease in the last 6 months. The local Ethics Committee has previously approved the study protocol, and all participants gave written informed consent.

### 2.1. Blood Pressure Measurements

Office blood pressure was obtained using an electronic device (model HEM-705CP, Omron Healthcare Inc., IL, USA) and an appropriate sized cuff. Patients were seated for 30 minutes before measurement and refrained from smoking and caffeine ingestion in this period of time. Three readings, one minute apart, were done, and the average of these measurements was defined as the patient clinic blood pressure. The patients underwent 24-hour ABPM in nondominant arm with SpaceLabs 90207 monitor (Spacelabs Inc., Redmond, WA, USA), validated by the British Hypertension Society and the Association for the Advancement of Medical Instrumentation protocol [24]. Readings were taken every 20 minutes during the day and every 30 minutes at night. The patients recorded their sleep and wake times during the monitoring. ABPM was considered adequate if >70% of measurements were successfully obtained. The percentage decline in nocturnal blood pressure was calculated as follows for systolic (SBP) and diastolic (DBP) blood pressures: percentage decline in nocturnal blood pressure = (daytime blood pressure−night-time blood pressure) ∗ 100/daytime blood pressure. The AASI was calculated from 1 minus the regression slope of diastolic pressure on systolic blood pressure. The slope was not forced through the origin.

### 2.2. Blood and Urine Samples Collection

 Fasting venous blood was collected from participants to measure total cholesterol, triglycerides (TG), high-density lipoprotein cholesterol (HDL-C), glucose, creatinine (enzymatic method), and uric acid. The low-density lipoprotein cholesterol (LDL-C) level was calculated by the Friedewald formula (8). eGFR was assessed by modified MDRD equation: eGFR = 0.741 × 175 × Cr^ −1.154^ × age^−0.203^(× 0.742 if female). C-reactive protein (nephelometry, BN II, Siemens AG Inc, Munich, Germany) and morning urinary spot albumin and creatinine (nephelometry, Immage, Beckman Coulter Inc, Fullerton, CA, USA) were also measured. The lower detection limit for C-reactive protein was 0.20 mg/L. Values for microalbuminuria were considered normal up to 30 mg/g creatinine.

### 2.3. Statistical Analyses

Data are presented as mean ± standard error of mean (SEM). For database management and statistical analyses, we used GraphPad Prism software, version 5.0 (GraphPad Software Inc., CA, USA). Chi-squared test (for 2 × 3 tables) or Fisher's exact test (for 2 × 2 tables) and Student's *t*-test were used to compare proportions and means, respectively. Pearson's correlation coefficients were used to explore associations between examined continuous variables with parametric distribution. Statistical significance was determined by *α*-level of 0.05 on two-sided tests.

## 3. Results

Baseline clinical characteristics did not differ between the groups. As expected, hemoglobin was significantly lower and serum creatinine and uric acid were significantly higher in CKD group (Table 1). Mean eGFR by MDRD in the CKD group was 35.3 ± 2.8 mL/min. C-reactive protein and UACR were significantly higher in CKD group (Table 1).

The mean office and ambulatory blood pressure readings were similar in both groups (Table 2), although CKD patients needed to use more antihypertensive drugs (2.7 ± 0.2 versus 2.2 ± 0.1, *P* = 0.0398) to obtain blood pressure control. When CKD was compared to SAH group concerning antihypertensive treatment, there was no significant difference for diuretics (60% versus 70%), angiotensin converting enzyme inhibitors/angiotensin receptor blockers (86% versus 77%) and beta blockers (40% versus 37%). However, use of calcium channel antagonists was significantly more common in CKD patients (47% versus 23%, *P* < 0.01).

**Figure 1:**
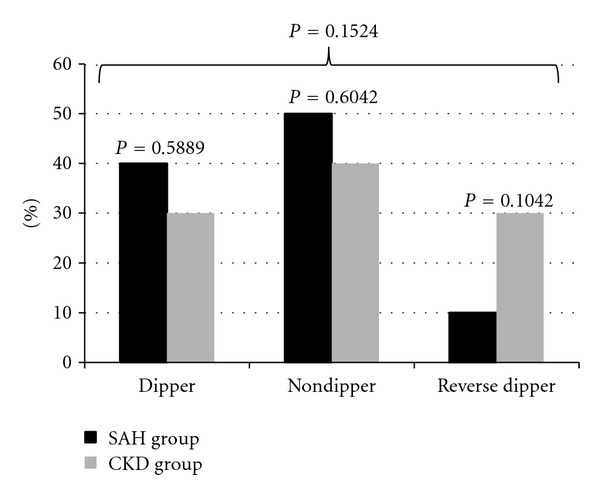
Percentage of nocturnal blood pressure fall patterns as dipper, nondipper, and reverse dipper in chronic kidney disease (CKD) and systemic arterial hypertension (SAH) groups.

The mean nocturnal systolic blood pressure fall was lower than 10% in both groups (4.0 ± 1.5% in CKD versus 7.6 ± 1.1% in SAH, *P* = 0.0588). There were 40% of dipper patients, 50% of nondipper, and 10% of reverse dipper in SAH group. On the other hand, CKD group presented 30% of dipping pattern, 40% nondipping, and 30% reverse dipping (Figure 1). Pulse pressure was not different between CKD and SAH groups (54 ± 2 versus 50 ± 2 mmHg, *P* = 0.20).

**Figure 2:**
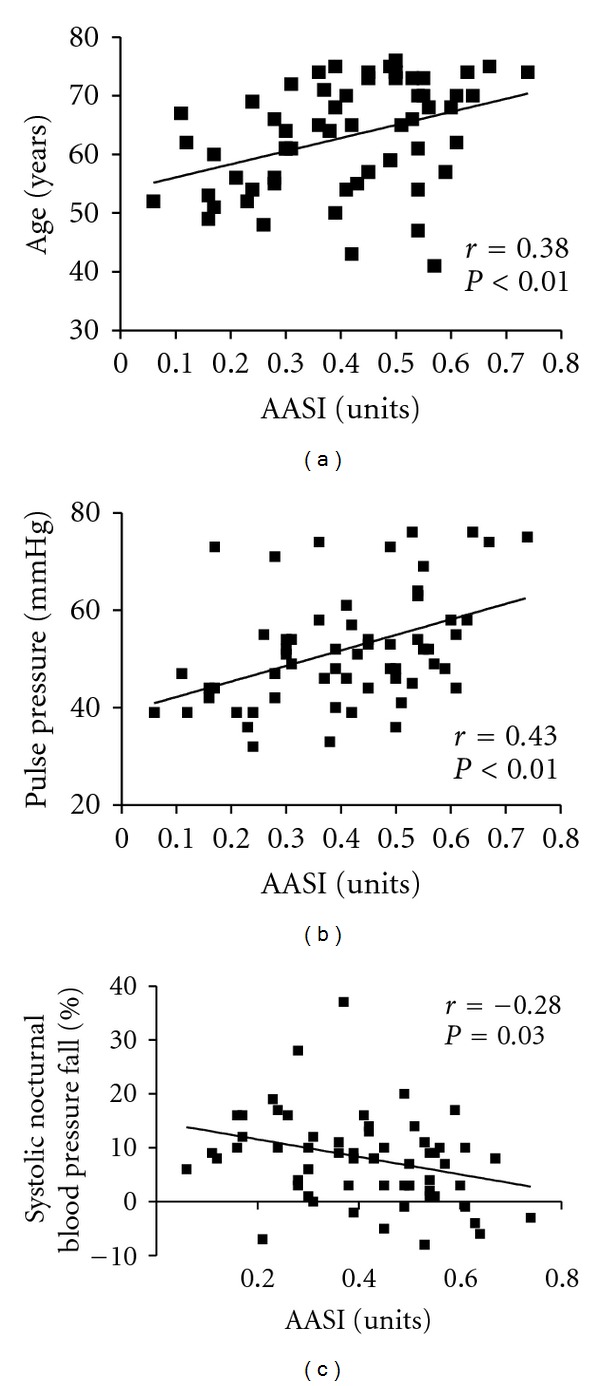
Plots of ambulatory arterial stiffness index on age (a), pulse pressure (b), and systolic nocturnal blood pressure fall (c).

**Figure 3:**
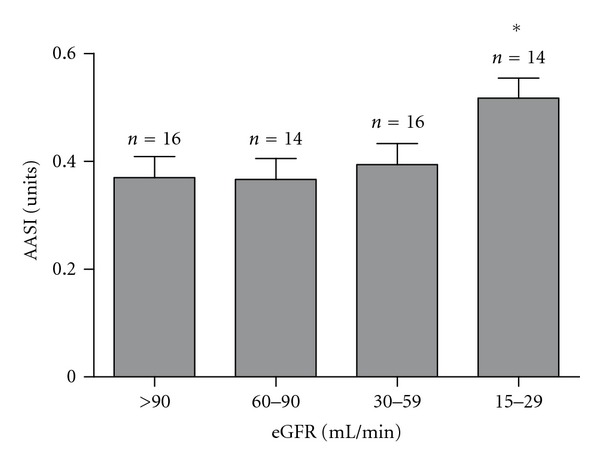
Ambulatory arterial stiffness index (AASI) according to estimated glomerular filtration rates (eGFR). Data are presented as mean ± SEM. *P < 0.05 versus other groups.

The AASI index was significantly higher in CKD patients when compared with SAH group (0.45 ± 0.03 versus 0.37 ± 0.02, *P* = 0.04). Correlation tests showed that AASI was positively related to age (*r* = 0.38, *P* < 0.01), pulse pressure (*r* = 0.43, *P* < 0.01) and inversely related to nocturnal blood pressure fall (*r* = −0.28, *P* = 0.03) (Figure 2). AASI did not correlate to UACR, serum creatinine, or eGFR. However, when eGFR was analyzed among all patients, those with eGFR less than 30 mL/min had higher AASI (Figure 3).

## 4. Discussion

The results of our study demonstrate that hypertensive patients with CKD presented a higher AASI when compared to those with normal renal function. Supporting the concept that AASI is a marker of arterial stiffness, Li et al. described its correlation with pulse wave velocity, central and peripheral augmentation indexes [15]. Moreover, in a cohort of 11,291 patients, Dolan et al. showed that AASI carried prognostic information, as it was a predictor of stroke and cardiac death [16]. In a Japanese study, Kikuya et al. also observed that AASI predicted cardiovascular and stroke mortality over and beyond pulse pressure [25]. Muxfeldt et al. demonstrated that AASI is a predictor of cardiovascular morbidity and mortality in 547 patients with resistant hypertension [21]. Furthermore, some research groups have already reported that AASI presents good reproducibility, with repeatability coefficients close to 60% [26, 27]. Criticizers say that this index is dependent on pulse pressure and dipping pattern and do not provide new information [28, 29]. Schillaci et al. studied 515 untreated hypertensive patients and found that AASI was strongly dependent on the degree of nocturnal blood pressure fall and only weakly related to pulse wave velocity [28]. Similar results were found by Baumann et al. with 112 German hypertensive patients [29].

Some authors have studied the relationship between AASI and renal function among hypertensive patients. Ratto et al. showed that AASI was positively related to urinary albumin excretion and negatively related to estimated creatinine clearance in a population of 168 patients with recently diagnosed hypertension and without drug treatment [30]. Mulè et al. studied 142 hypertensive patients without drug treatment and with serum creatinine less than 1.5 mg/dL and demonstrated that patients with high AASI presented lower GFR [17]. This paper also suggested that AASI was a better predictor of GFR decline than 24 h pulse pressure. In 554 hypertensive patients with and without drug treatment, Garcia-Garcia et al. observed that AASI correlates with eGFR, carotid intima-media thickness, and Cornell voltage-duration product [20].

Ageing is an important factor for arterial stiffening. Elderly people are predisposed to lose arterial elastic laminae and increase collagen deposits in vascular wall [10, 31]. This way, assuming AASI as a marker of vascular stiffness, the relationship between AASI and age is expected. Interestingly, CKD patients tend to have stiffer vessels when compared to age- and blood-pressure-matched patients with normal renal function [31, 32]. Beyond traditional risk factors, such as hypertension and dyslipidemia, uremia seems also play a role to this finding. Mineral metabolism alterations and arterial calcification are probably relevant mechanisms [10, 33]. This may be one hypothesis to explain why AASI was higher in CKD than in SAH group despite similar office and ambulatory blood pressure measurements in the present study. Moreover, patients with the lowest eGFR (stage 4, according to the American National Kidney Foundation [4]) presented higher AASI.

Pulse pressure and nocturnal blood pressure fall are two parameters from ABPM that correlate with arterial stiffness [34, 35]. Lekakis et al. and Jerrard et al. showed that hypertensive patients with nondipper pattern presented stiffer vessels when measured by pulse wave velocity, suggesting a relationship between blunted nocturnal blood pressure fall and reduced arterial elasticity [35, 36]. These findings corroborate with our study, since nocturnal blood pressure fall and pulse pressure were correlated with AASI. Indeed, AASI was different between groups despite similar pulse pressure and dipper status. This emphasizes the importance of calculating AASI after ABPM.

The present study has limitations considering the small sample size. Moreover, it was not prospective and not focused on clinical outcomes. However, our data strongly implies the value of AASI as a noninvasive tool for hemodynamic evaluation of CKD patient and reinforces the role of ABPM in hypertensive patients with renal dysfunction. Increased AASI might be one of the pathophysiological changes observed in CKD patients before the progression to end stage renal disease. More studies are needed to support the clinical usefulness of this parameter, but we propose that the software for ABPM analysis should include AASI value as a marker of cardiovascular risk assessment in the near future.

## Figures and Tables

**Table 1:** Demographic, anthropometric, and laboratory data of hypertensive patients in both groups.

Variable	SAH group	CKD group	*P *value
Age (years)	62.8 ± 1.7	63.2 ± 1.7	0.8786
Men, *n* (%)	18 (60)	18 (60)	1.0000
Black, *n* (%)	4 (13)	7 (23)	0.3251
Current smokers, *n* (%)	5 (17)	4 (13)	0.7232
Previous cerebrovascular disease, *n* (%)	2 (7)	2 (7)	1.0000
Dyslipidemia, *n* (%)	14 (47)	13 (43)	0.7091
BMI (kg/m^2^)	27.4 ± 0.8	26.4 ± 0.8	0.3856
Waist-to-hip ratio	0.93 ± 0.01	0.92 ± 0.01	0.6881
Hemoglobin (g/dL)	13.9 ± 0.2	12.5 ± 0.2	<0.0001
Glucose (mg/dL)	95.8 ± 11.1	98.7 ± 8.9	0.2645
Creatinine (mg/dL)	0.82 ± 0.17	2.26 ± 0.78	<0.0001
eGFR (mL/min)	92.8 ± 4.8	35.3 ± 2.8	<0.0001
Sodium (mg/dL)	139 ± 0.4	137 ± 3.4	0.6317
Potassium (mg/dL)	4.3 ± 0.09	4.8 ± 0.08	0.0927
Uric acid (mg/dL)	6.0 ± 2.0	8.4 ± 1.8	<0.0001
Triglycerides (mg/dL)	164 ± 100	232 ± 149	0.0737
Total Cholesterol (mg/dL)	205 ± 36	200 ± 41	0.6355
LDL-cholesterol (mg/dL)	120 ± 39	106 ± 33	0.1489
HDL-cholesterol (mg/dL)	51 ± 24	39 ± 10	0.0199
C-Reactive protein (mg/L)	2.6 ± 0.6	6.4 ± 1.7	0.0338
UACR (mg/g)	19 ± 5	367 ± 90	0.0002
LVH in ECG, *n* (%)	1 (4)	4 (13)	0.2216

Data presented as mean ± SEM or *n* (%). SAH, systemic arterial hypertension; CKD, chronic kidney disease; BMI, body mass index; eGFR, estimated glomerular filtration rate by MDRD equation; LDL, low-density lipoprotein; HDL, high-density lipoprotein; UACR, urinary albumin-creatinine ratio; LVH, left ventricular hypertrophy; ECG, electrocardiogram.

**Table 2:** Office and ambulatory blood pressure parameters of hypertensive patients with normal and impaired renal function.

BP parameters	SAH group	CKD group	*P* value
AASI, units	0.37 ± 0.02	0.45 ± 0.03	0.0400
Office systolic BP, mmHg	149 ± 3	145 ± 4	0.4452
Office diastolic BP, mmHg	87 ± 2	85 ± 2	0.5121
Controlled office BP, *n* (%)	15 (50)	10 (33)	0.2949
24 h systolic BP, mmHg	131 ± 3	133 ± 3	0.6172
24 h diastolic BP, mmHg	81 ± 2	79 ± 2	0.6004
24 h Pulse Pressure, mmHg	50 ± 2	54 ± 2	0.2034
Controlled 24 h BP, *n* (%)	14 (47)	14 (47)	1.0000
White coat effect, *n* (%)	6 (20)	3 (10)	0.2859
Daytime systolic BP, mmHg	134 ± 3	135 ± 3	0.8732
Daytime diastolic BP, mmHg	83 ± 2	81 ± 2	0.5507
Nocturnal systolic BP, mmHg	124 ± 3	129 ± 3	0.2707
Nocturnal diastolic BP, mmHg	75 ± 2	74 ± 2	0.9644
Systolic nocturnal fall, %	7.6 ± 1.1	4.0 ± 1.5	0.0588
Diastolic nocturnal fall, %	10.7 ± 0.1	8.6 ± 0.2	0.2945

Data are expressed as mean ± SEM or *n* (%). AASI, ambulatory arterial stiffness index; SAH, systemic arterial hypertension; CKD, chronic kidney disease; BP, blood pressure.
